# Prevalence of Pulmonary Tuberculosis among Prison Inmates in Ethiopia, a Cross-Sectional Study

**DOI:** 10.1371/journal.pone.0144040

**Published:** 2015-12-07

**Authors:** Solomon Ali, Abraham Haileamlak, Andreas Wieser, Michael Pritsch, Norbert Heinrich, Thomas Loscher, Michael Hoelscher, Andrea Rachow

**Affiliations:** 1 College of Health Sciences, Jimma University, Jimma City, Ethiopia; 2 Division of Infectious Diseases and Tropical Medicine, Medical Centre of the University of Munich (LMU), Munich, Germany; 3 German Centre for Infection Research (DZIF), partner site Munich, Germany; 4 CIH^LMU^ Center for International Health, Ludwig-Maximilians-Universität, Munich, Germany; Hebrew University, ISRAEL

## Abstract

**Setting:**

Tuberculosis (TB) is one of the major health problems in prisons.

**Objective:**

This study was done to assess the prevalence and determinants of active tuberculosis in Ethiopian prisons.

**Design:**

A cross-sectional study was conducted from January 2013 to December 2013 in 13 zonal prisons. All incarcerated inmates underwent TB symptom screening according to WHO criteria. From identified TB-suspects two sputum samples were analyzed using smear microscopy and solid culture. A standardized questionnaire assessing TB risk factors was completed for each TB suspect.

**Results:**

765 (4.9%) TB suspects were identified among 15,495 inmates. 51 suspects were already on anti-TB treatment (6.67%) and 20 (2.8%) new culture-confirmed TB cases were identified in the study, resulting in an overall TB prevalence of 458.1/100,000 (95%CI: 350-560/100,000). Risk factors for active TB were alcohol consumption, contact with a TB case before incarceration and no window in prison cell. HIV prevalence was not different between TB suspects and active TB cases. Further, the TB burden in prisons increased with advancing distance from the capital Addis Ababa.

**Conclusions:**

The overall TB prevalence in Ethiopian prisons was high and extremely variable among different prisons. TB risk factors related to conditions of prison facilities and the impact of implemented TB control measures need to be further studied in order to improve TB control among inmates.

## Introduction

Tuberculosis (TB) is a major health problem in prisons and its prevalence was reported to be multiple times higher compared to that of the general population. Conditions such as overcrowding, malnutrition and limited access to medical care which often exist in prisons increase the risk of reactivation, transmission and poor prognosis of tuberculosis disease among inmates [1,2].

Several cross-sectional studies estimated the prevalence of TB in African prisons. Studies published from Cameroon, Zambia and Malawi reported relatively differing prevalence which was between 2.6 and 10 times higher than in the general population of the respective country [1,3,4]. From Ethiopia, so far three studies were reported from 5 different prisons in Eastern Ethiopia, North Gondar zone and Gamo Gofa zone. The reported numbers for TB prevalence, 1,913, 1,482.3 and 629 TB cases per 100.000 inmates respectively, were comparable to that from other African settings [5–7].

Various factors were specified as determinants of tuberculosis in prisons. Among them low socio economic status, history of anti TB treatment before incarceration, previous contact with TB patients, low body mass index and HIV infection were frequently associated with active TB in prisons in different studies [1,5,6,8,9].

Prisons are regulated but not closed systems, due to the numbers of people who constantly enter, leave and re-enter into them. Therefore, prison health is a critical part of public health as health problems within and outside prisons are interrelated [10]. Every successful TB control program also requires effective TB control in prisons and failure to control TB in prisons has the potential to disrupt community TB control programs [11]. Ethiopia is one of the 22 tuberculosis high burden countries with a recent TB prevalence of 211 per 100,000 populations [12]. According to the Ethiopian Human Right commission 2012 report, a total of 86,610 inmates were incarcerated in 119 prisons in Ethiopia [13].

Despite this fact, relatively little attention has been given to assess the condition of TB in Ethiopian prisons in the past. Those 3 studies mentioned above [5–7] were relatively small and limited to 3 areas of the country. To the best of the author’s knowledge, this is the first huge study conducted in more than 15,000 inmates of 13 prisons in three different regional states of Ethiopia to systematically determine the prevalence of TB and its risk factors in Ethiopian prisons.

## Materials and Methods

### Study setting

Ethiopia is administratively organized within nine regions and two federal cities. Among these, Oromia, Amhara and Southern Nations, Nationalities and Peoples Regional State (SNNPRS) are the three biggest regions with a total population of approximately 67,730,002 (more than 80% of total Ethiopian population). Harari is the smallest regional state with a population of only 210,000 [14]. Due to logistic reasons such as cooperation with investigators and accessibility from the study centre at Jimma University the participating prisons for this study were selected from Oromia, SNNPRS and Harari regional states. These three regional states together cover an area where almost 60% of the total Ethiopian population resides [14]. Oromia regional state had 37 (17 zonal and 20 district) prisons, while SNNPRS had 23 (13 zonal and 10 district) prisons. Harari regional state had only one zonal prison [13]. Zonal administrative prisons are the largest prisons in Ethiopian context. A total of 13 zonal administrative level prisons were randomly selected by lottery method. Accordingly, seven out of 17 and five out of 13 prisons were drawn from Oromia and SNNPRS, respectively, while the only prison of Harari regional state was included in the study. By this approach, ca. 35% of the total prison population of the included regional states was represented in this study. All zonal prisons had a small clinic which was equipped to handle emergency situations and to treat frequent infections with antibiotics. For (microbiological) diagnosis and treatment of more complicated, severe and chronic diseases, including TB, inmates were referred to nearby hospitals or health facilities. In diagnosed TB patients treatment was provided and supervised by prison clinics according to national guidelines.

### Data and sample collection

A cross sectional study was done from January 2013 to December 2013. Study activities were conducted in one prison after the other in the following order: Jimma, Nekemte, Ambo, Wolkite, Shashemene, Asella, Bonga, Mizan, Yabelo, Dilla, Sodo, Asebeteferi/Chiro, Harar prison. Prison inmate health committee members together with health professionals working in prison were trained on scientific purposes, ethical aspects and data collection procedures of this study.

In a first step, all inmates were registered with support of the health committee members in each prison. Then TB-symptom screening was conducted by the research staffs using a questionnaire provided by WHO [15]. All inmates who were 18 years or older and who fulfilled at least one of three screening criteria listed below were considered as TB suspects and included in the study [15].

1. Inmates with a score of 5 according to WHO recommended tuberculosis suspect identification criteria: Cough of two weeks duration (scored as 0 or 2), sputum production (scored as 0 or 2), chest pain (scored as 0 or 1), recent loss of appetite (scored as 0 or 1) and loss of weight in last 3 months (scored as 0 or 1).
2. Inmates who had history of anti TB treatment in the past five years.
3. Inmates living with HIV.

Each study participant was examined and interviewed by a study clinician using a pre-designed questionnaire (S1 File). All study participants were counseled and tested for HIV in line with the Ethiopian national algorism for HIV testing and counseling. Two early morning sputum samples were collected from each participant on two consecutive days, after instructions about technique of coughing and sputum quality were provided by study staff. The quality of sputum was checked upon reception, saliva and soil contaminated specimens were rejected and participants were asked to bring another specimen. The first early morning sputum was stored at 2–8^°^C for a maximum of one week until transported to Jimma University Mycobacteriology Laboratory where sputum culture was performed. The second early morning sputum was processed in prisons for immediate diagnosis of tuberculosis by smear-microscopy after Ziehl-Neelsen staining and was then re-read by an experienced microbiologist in Jimma. Completeness and accuracy of all study documents, including the questionnaire, screening logs and laboratory log books, were checked daily by the local principal investigator. Sputum received in Jimma University Mycobacteriology laboratory was processed using Specimen Digestion/Decontamination Kit following the manufacturer´s instructions [16]. The processed samples were inoculated on LJ slants (Lowenstein-Jensen Medium) as described by the manufacturer [17], including growth control using H37RV strain in 5% of LJ-slopes and sterility control for all used slopes. Apart from manufacturer instructions all assays were performed according to the implemented standardized operating procedures.

### Data analysis

All data were recorded on standardized data collection forms. Data were double-entered in an excel data base. Analysis was done by STATA version 10. In the descriptive analysis, for categorical variables proportions with 95%CI and for continuous variables means with 95%CI were calculated. Univariable logistic regression analysis was conducted to analyze the association between individual risk factors and TB-diagnosis; a p-value of <0.05 was considered as significant. Factors with significant association were used to build the final multivariable logistic regression model using a forward elimination approach. Likelihood ratio test was performed to confirm significant association of each risk factor with the outcome in multivariable regression model and to test for a linear trend for categorical variables.

### Ethical considerations

Ethical clearance was obtained from Jimma University Ethical Review Board. Written consent was sought from each study participant. Directly observed treatment short course was started for newly diagnosed TB patients in collaboration with the nearby health center. Newly diagnosed HIV positive participants were linked to the nearby health institutions for follow up and possible initiation of anti-retroviral therapy. Permission to conduct the research was granted by relevant prison authorities.

## Results

**Fig 1:**
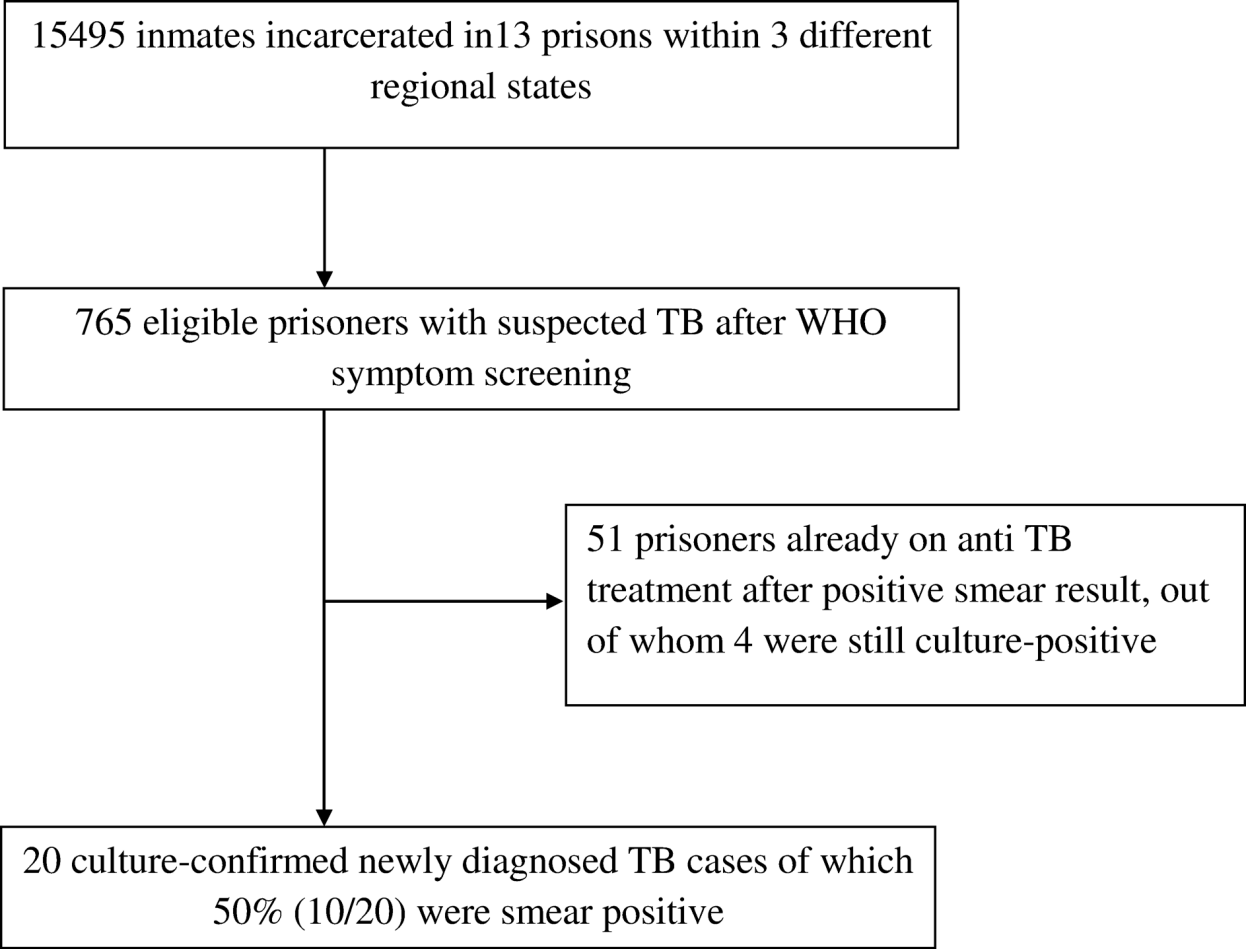
Overall study flow. Total prisons included, inmates screened, identified suspects and detected TB cases were presented by absolute number.

In this study, a total of 15,495 inmates incarcerated in 13 different prisons underwent TB symptom screening. Seven hundred sixty five (4.9%), (95%CI: 4.6%-5.2%) fulfilled the TB screening criteria out of whom 51 (6.7%) were already diagnosed earlier by Ziehl-Neelsen smear microscopy and placed on anti tuberculosis treatment during incarceration. Among the remaining 714 participants 20 (2.8%) were newly diagnosed with active pulmonary tuberculosis in this study. Ten (50%) of them were positive by smear microscopy. Thus, TB prevalence among suspects was 9.2% (71/765), (95%CI: 7.2–11.4), and among all prisoners it was 0.46% (71/15,495), (95%CI: 0.35–0.57) (Fig 1)

### Characteristics of study participants

Out of the 765 participants, 96.8% were male (Table 1). The mean age was 32.5 (95%CI: 31.5–33.4). 4.44% (34/765) of participants were tested positive for HIV. Three of them were also diagnosed with active TB, resulting in a HIV-prevalence of 4.23% (3/71) among TB cases. The majority (68.5%) were farmers before incarceration. Approximately two thirds were following Muslim (38.6%) or Orthodox (30.7%) religions. Most participants (66.9%) were married. More than one third (39.1%) were either illiterate or had no formal education (Table 1). The mean duration of stay in prison was 26.1 months (95%CI: 24.1–28.1) at the time point when the study was conducted, with no difference between TB suspects and confirmed TB cases. Eighty eight percent of participating inmates had no history of incarceration before the current sentence.

**Table 1:** Socio demographic characteristics and TB risk factors of participants.

Characteristic		Total N = 765	Proportion in % (95%CI)
**Sex**			
	Male	741	96.86 (95.6–98.1)
**Age**			
	≤ 45 Years	651	85.1 (82.6–87.6)
**Religion**			
	Muslim	295	38.61 (35.2–42.1)
	Orthodox	235	30.76 (27.5–34.0)
	Protestant	200	26.18 (23.1–29.3)
	Ahizab	18	2.36 (1.3–3.4)
	Catholic	6	0.79 (0.2–1.4)
	Others	10	1.31 (0.5–2.1)
**Occupation**			
	Farmer	524	68.5 (65.2–71.8)
	Student	86	11.24 (9.0–13.5)
	Merchant	42	5.49 (3.9–7.1)
	Employee	30	3.92 (2.5–5.3)
	No job	10	1.31 (0.5–2.1)
	Driver	7	0.92 (0.2–1.5)
	Others	66	8.63 (6.6–10.6)
**Marital Status**			
	Married	512	66.93 (63.6–70.2)
	Single	229	29.93 (26.7–33.2)
	Divorced	18	2.35 (1.3–3.4)
	Widowed	6	0.78 (0.2–1.4)
**Education**			
	Illiterate	286	37.39 (33.9–40.8)
	Read & write	13	1.70 (0.8–2.6)
	1–4 grade	157	20.52 (17.7–23.4)
	5–8 grade	208	27.19 (24.0–30.3)
	9–12 grade	83	10.85 (8.6–13.1)
	>12 grade	18	2.35 (1.3–3.4)
**History of incarceration**			
	No	678	88.63 (86.4–90.1)
**HIV serology**			
	**Negative**	**731**	**95.56 (94.1–97.0)**

### Variability of TB-prevalence among different prisons

Considering the 51 already existing and the 20 newly diagnosed TB cases, the overall point prevalence of tuberculosis in these 13 prisons was 458.1 (95%CI:350–560) per 100,000 inmates, though there was great variability among prisons (Table 2). The highest TB prevalence was observed in Dilla prison (SNNPRS) with 1528 cases per 100,000 inmates. Opposed to that, there was no TB case detected at Wolkite (SNNPRS) and Asebeteferi/Chiro prisons (Oromia). The point prevalence of newly diagnosed TB was 129 (95%CI: 70–190) per 100,000 inmates with a variability that ranged from no new TB case detected in five prisons to 887.6 new TB cases per 100,000 which was observed at Yabelo prison (Oromia).

**Table 2:** Prison characteristics and prevalence of TB by prison.

Prison	Total inmates	Total area (m^2^)	Inmates/m^2^	Prison distance from Addis Ababa in km	TB suspects identified (%)	Prisoners already on TB treatment (%)*	Newly diagnosed TB cases (%)*	Smear positives among new cases (%)*	Prevalence newly diagnosed TB cases per 10^5^	Prevalence all TB cases per 10^5^
**Ambo** ^**1**^	1602	1413	1.13	126	45 (2.8)	1 (2.2)	-	-	-	62.4
**Asebeteferi** ^**1**^	1243	942	1.32	326	28 (2.3)	-	-	-	-	-
**Asella** ^**1**^	1067	2270	0.47	175	53 (5.0)	1 (1.9)	1(1.9)	0 (0)	93.7	187.4
**Bonga** ^**2**^	1306	nd	nd	465	58 (4.4)	14 (24.1)	1(2.3)	1(2.3)	76.6	1148.5
**Dilla** ^**2**^	916	759.50	1.21	405	58 (6.3)	8 (13.8)	6 (12.0)	3 (50.0)	655.0	1528.4
**Harar** ^**3**^	1511	1944.24	0.78	526	60 (4.0)	7 (11.7)	1(1.9)	0 (0)	66.2	529.5
**Jimma** ^**1**^	1267	2133	0.59	355	140 (11.0)	4 (2.8)	-	-	-	315.7
**Mizan** ^**2**^	1929	1505	1.28	561	59 (3.0)	2 (3.4)	2 (3.5)	1 (1.7)	103.7	207.4
**Nekemte** ^**1**^	1172	nd	nd	328	70 (6.0)	7 (10.0)	-	-	-	595.7
**Shashemene** ^**1**^	1139	1426	0.80	254	83 (7.3)	2 (2.4)	1(1.2)	0 (0)	87.8	263.4
**Sodo** ^**2**^	1274	1294	0.98	383	38 (3.0)	1 (2.6)	2 (5.4)	2 (5.4)	157.0	235.5
**Wolkite** ^**2**^	393	807.50	0.49	155	16 (4.1)	-	-	-	-	-
**Yabelo** ^**1**^	676	819	0.83	570	57(8.4)	4 (7.0)	6 (11.3)	3 (5.6)	887.6	1479.3
**Total N/Mean**	**15495**	**15313.2**	**0.85**	**356.07**	**765 (4.9)**	**51 (6.67)**	**20 (2.8)**	**10 (1.4)**	**129.0**	**458.1**

%: percentage, m^2^: square meters, km: kilometers, nd: no data, SNNRS: south nations and nationalities regional state

* among identified TB suspects

^1^ Oromia Region

^2^ SNNPRS

^3^ Harare Region

Among the different regional states the SNNPRS had the highest tuberculosis burden with an overall prevalence of 618.8 (95%CI: 420–820) per 100,000 inmates. The prevalence of TB in Harar and Oromia regional states were 529.5 (95%CI: 160–590) and 330.5 (95%CI: 210–460) per 100,000 inmates, respectively. We found a linear trend in prevalence of tuberculosis with advancing distance of the prisons from the centre of Ethiopia (Addis Ababa). Prisons within a radius of below 200km distance from Addis Ababa had the lowest TB prevalence of 97.98 (95%CI: 10–210) per 100,000 inmates while the highest TB prevalence of 804 (95% C.I: 580–1020) per 100,000 inmates was observed in prisons located more than 400km away from Addis Ababa (S1 Fig), (OR = 3.60, 95% CI: 2.24–5.70, p<0.0001).

In all prisons, the mean number of incarcerated inmates per cell was 134.8 (95%CI: 129.2–140.3). In prisons like Ambo, Asebeteferi/Chiro, Dilla and Mizan the number of inmates incarcerated per square meter area was greater than one. The lowest number of inmates incarcerated per square meter was seen in Wolkitie with around 0.5. However there was no significant association found between the number of inmates per square meter and TB prevalence in prisons (OR = 1.59, 95%CI: 0.97–2.59, p = 0.07).

### Risk factors associated with active TB

In this study, 71 smear or culture confirmed TB cases were compared with 688 inmates without TB to identify risk factors for active TB disease. Alcohol consumption and history of contact with TB patient at home were significantly associated with active TB disease, while the availability of a window in the prison cell reduced the probability of TB in prisoners (Table 3). In our study population, risk factors such as education level, mean duration of stay in prison, cigarette smoking, chewing khat and positive HIV-status were not significantly associated with TB disease (Table 3).

**Table 3:** Logistic regression analysis of risk factors for active TB disease in prisoners.

Variable	Univariable analysis*		Multivariable analysis*	
	COR (95%CI)	p-value	AOR (95%CI)	p-value
Incarcerated in cell with window	0.25 (0.15–0.42)	<0.001	0.26 (0.16–0.45)	<0.001
Alcohol consumption	1.98 (1.20–3.21)	0.008	2.04 (1.20–3.46)	0.008
TB case contact at home	1.59 (1.18–2.15)	0.002	1.49 (1.08–2.06)	0.02
TB case contact in prison	1.15 (0.82–1.61)	0.41	-	-
Religion	1.09 (0.91–1.30)	0.37	-	-
Education	0.93 (0.79–1.09)	0.35	-	-
Duration of stay in prison	1.06 (0.86–1.32)	0.58	-	-
Cigarette smoking	0.93 (0.73–1.17)	0.53	-	-
Khat chewing	1.12 (0.84–1.51)	0.44	-	-
Positive HIV serology	0.9 (0.28–3.14)	0.91	-	-

COR: crude odds ratio, AOR: adjusted odds ratio, CI: confidence interval

*Six participants without active TB disease were removed from analysis due to incomplete data set

## Discussion

Recently three studies were published on the prevalence of tuberculosis in Ethiopian prisons. However, these studies included only five prisons in total which covered an average of 2.2%, 3.6% and 4.4% of total inmates incarcerated at that time in Ethiopia, respectively [5–7]. To the best of our knowledge, this is the first large scale study performed in 13 prisons located in the south western, southern and eastern part of the country which covered about 18% of inmates incarcerated in whole Ethiopia.

In this study the overall prevalence of TB was 458.1 per 100,000 inmates. In comparison to data published by WHO in 2014 the observed prevalence in our prison study was still more than two times higher than that estimated for the general Ethiopian population which was 211 per 100,000[12]. It is not a surprise to see such an increment of TB considering conditions such as overcrowding, which could also be observed in some prisons in this study, as well as nutritional factors and limited access to medical care existing in prisons, On the other hand, the observed overall TB prevalence in this study was much lower than reported from the previous Ethiopian studies [5–7], conducted in 2008 to 2012. Possible reasons for that might be differences in study size, a low sensitivity of the relatively strict inclusion criteria applied in our study and the low HIV-prevalence of 4.4%. The lower TB prevalence in our prison study might be also associated with the decline of TB prevalence in the general population observed for Ethiopia in the last five years. The national TB survey of 2010/2011 reported a TB prevalence of 277/100000 [18], while in 2013 the prevalence of TB in the general population was declined to 211/100000 [12]. Further, Ethiopia is among those African countries which had achieved the 2015 global targets announced by the Stop TB partnership, reflecting the efforts of the national government and its allies to control TB in the country, including TB in prisons.

Interestingly, we observed a great variability of TB prevalence among different prisons, varying from no TB case detected in two prisons while 1,528.4 TB cases per 100,000 prisoners found in a prison with the highest TB prevalence. The data suggest that there might be relevant differences in the efficiency and commitment of the prison health workers or responsible authorities to implement systematic and effective TB-control strategies. For instance, in some prisons facilities was no segregation area for newly diagnosed or infectious TB patient inmates. Further, inmates incarcerated in rooms without a window had a four times higher TB risk than those incarcerated in rooms where a window was present. Other prison studies from Ethiopia and Thailand which assessed the effect of ventilation through windows observed similar findings [5,8]. On the other hand, crowding measured as number of inmates per square meter of prison cell was not associated with TB diagnosis in this study. Of note, only 28.2% of all prevalent TB cases were newly diagnosed in this study. The remaining cases were already on TB-treatment after positive smear-microscopy result. This indicates that in some studied prisons the implemented strategies for TB-detection and treatment were effectively installed. Additionally, distance from the capital Addis Ababa might play a role in the occurrence of TB in prisons. This finding is interesting and cannot be explained by greater negligence of TB in more remote prisons only as the number of both, already treated cases and newly diagnosed cases, were equally increased in peripheral prisons. As inmates who reported a previous TB-contact at home or alcohol consumption had a significant higher risk to be diagnosed with TB one could speculate that the higher TB prevalence in remote prisons also relates to certain risk behavior and consequently a relatively higher TB burden in certain sub-groups of the general population in remote areas. A decreased awareness of tuberculosis and its transmission which might be associated with illiteracy and lower socio-economic standard, a poorer health system infrastructure including the lack of well-trained health professionals and the geo-climatic conditions one can find in remote areas of southern Ethiopia might also explain the observed differences in TB-prevalence among the prisons in this study.

HIV-infection was not an independent risk factor for TB-infection in our study. However, this could be most likely explained by the comparably low HIV-prevalence in those prisons which were included in this study compared to other publications [9,19,20].

Although performed in a large number of prisoners our study has several shortcomings which demand that the results are interpreted with caution. First, the relatively strict inclusion criteria for symptomatic prisoners which demand productive cough for at least 2 weeks plus one additional TB symptom might have resulted in an underestimation of TB-prevalence. This assumption might be supported by the low proportion (4.9%) of TB suspects in this study compared to other publications [5,6]. On the other hand, in most settings the symptom-based WHO screening is the best tool available as extensive screening using microbiological tests or chest x-ray in all inmates are not affordable to many African countries. Further, the delay between sputum collection and processing for culture might have led to an increased number of samples without a positive result for *M*.*tb* due to growth of contaminating flora in 12.5% of cultures. Second, although the socio-demographic and risk factor questionnaire was translated into local languages and study staff was trained on its application bias due to over or under reporting of risk factors could still have occurred as prisoners might not remember facts correctly or did not want to reveal the true information. Third, our findings might not be representative for the prison population of the whole country due to restriction to prisons in only three regional states and the selection process of studied prisons. Given the high variability of TB prevalence and prison characteristics we observed among the included prisons one could speculate that there might be even a higher diversity among prisons across the whole country.

## Conclusion

The average TB prevalence in prison inmates is twice higher than the prevalence in the general population and a great variability of prevalence among different prisons existed. This variability and the higher TB burden in prisons located far from the capital suggest that the national TB control measures are either not similarly implemented in the different prisons or have a differing impact on TB-control in specific prison environments or study populations. This needs further attention and future studies should focus on risk factors related to the individual but also on factors inherent to the general population and the prison environment including the functioning of TB control strategies. Ongoing attention to those prisoners with specific TB risk factors such as TB contact at home, alcohol drinking and, still, with known positive HIV-status is demanded.

## Supporting Information

S1 File Questionnaire used for data collection. (PDF)

S1 Fig TB prevalence in prisons by distance from Addis Ababa. <200km from Addis Ababa: Ambo, Wolkite and Asella prisons, 200-400km from Addis Ababa: Shashemene, Nekemte, Sodo, Asebe Teferi/Chiro, Jimma´prisons, >400km from Addis Ababa: Bonga, Mizan, Dilla, Yabelo and Harar prison.
(TIF)
