# Supplementary material for: Prevalence of Pulmonary Tuberculosis among Prison Inmates in Ethiopia, a Cross-Sectional Study
Source: PLoS One. 2015 Dec 7;10(12):e0144040. doi: 10.1371/journal.pone.0144040 (PMC4671540; doi:10.1371/journal.pone.0144040)
Supplement: S1 File — (PDF) [file pone.0144040.s001.pdf]

Annex IIA Questionnaire for TB suspects

Name of prison______________ Prisons code: ___________Study ID __________

Date of data collection_______________ Name of interviewer: _________________________

Please read each question step by step to the participant and mark "X" on the box provided under response or write down the response as appropriate

Ser. Nº	Question to be asked	Proposed response	Coded response
Q01	Sex	□ Male<br>□ Female	0<br>1
Q02	Age [years]		
Q03	Address or home town, name of village before incarceration, no complete address	___________________	
Q04	Occupation before incarceration	□ Farmer<br>□ Merchant<br>□ Employed<br>□ Others: specify______	0<br>1<br>2<br>3
Q05	Educational status	□ Illiterate<br>□ Read and write only<br>□ 1-4 grade<br>□ 5-8 grade<br>□ 9-12 grade<br>□ Above 12	0<br>1<br>2<br>3<br>4<br>5
Q06	Ethnicity	□ Oromo<br>□ Amhara<br>□ Wolita<br>□ Somali<br>□ Sidama<br>□ Others: specify______	0<br>1<br>2<br>3<br>4<br>5
Q07	Religion	□ Orthodox<br>□ Muslim<br>□ Catholic<br>□ Protestant<br>□ Others : specify______	0<br>1<br>2<br>3<br>4
Q08	Marital status	□ Married<br>□ Single<br>□ Divorced<br>□ Widowed	0<br>1<br>2<br>3

Q09	Do you have cough for ≥ 2 weeks?	□ No<br>□ Yes	0<br>1
Q10	Do you have fever?	□ No<br>□ Yes	0<br>1
Q11	Do you have night sweats?	□ No<br>□ Yes	0<br>1
Q12	Do you have chest pain?	□ No<br>□ Yes	0<br>1
Q13	Do you have loss of appetite?	□ No<br>□ Yes	0<br>1
Q14	Do you have haemoptysis?	□ No<br>□ yes	0<br>1
Q15	Did you have loss of weight in the past 3 months?	□ No<br>□ yes	0<br>1
Q16	Did you have close contact with a person coughing for more than two weeks?	□ No<br>□ Yes	0<br>1
Q17	How often did you drink alcohol?	□ Never<br>□ ≤ 3 days per week<br>□ > 3 days per week	0<br>1<br>2
Q18	Which alcohol did you frequently drink	□ Areki(local)<br>□ Tela (local)<br>□ Borde(local)<br>□ Beer<br>□ Wine<br>□ Other, specify_____	0<br>1<br>2<br>3<br>4<br>5
Q19	How much cigarettes did you smoke?	□ None<br>□ Occasionally<br>□ One packet per week<br>□ More than one packet per week	0<br>1<br>2<br>3
Q20	How frequent do you chew "Chat"?	□ None<br>□ ≤ 1x per week<br>□ > 1x per week	0<br>1<br>2
Q21	Have you been imprisoned before?	□ No<br>□ Yes	0<br>1
Q22	How long have you been imprisoned in the current prison?	________ months	
Q23	Are you imprisoned with a known TB patient in the same room?	□ No<br>□ Yes<br>□ I don't know	0<br>1<br>2
Q24	In which block of the prison are you incarcerated?	_________	
Q25	How many inmates are imprisoned in your room?	________	

Q26	Do you have a window in your room?	□ No<br>□ Yes	0<br>1
Q27	If yes to Q. 26, how many?	________	
Q28	If yes to Q. 26 how often do you open the window?	□ Not at all<br>□ Half a day<br>□ Full day<br>□ Day and night	0<br>1<br>2<br>3
Q29	Have you been diagnosed for TB before this study?	□ No<br>□ Yes	0<br>1
Q30	If yes to Q. 29, when have you been diagnosed for TB?	□ Before imprisonment<br>□ During imprisonment	0<br>1
Q31	If yes to Q. 29, did you receive treatment?	□ No<br>□ Yes	0<br>1
Q32	If yes to Q. 29 did you complete the full course of treatment?	□ No<br>□ Yes<br>□ I don't know	0<br>1<br>2
Q33	Did you have contact with a known TB patient at home?	□ No<br>□ Yes<br>□ I don't know	0<br>1<br>2
Q34	Do you have BCG scar on your arm? Please check the scar	□ No<br>□ Yes	0<br>1

For participant;

We very much thank you for your time and appreciate your patience to respond to these questions.

For data collector;

Please proofread the questionnaire for completeness and hand over to the supervisor right after.
