# Supplementary figures and images for: Prevalence of Pulmonary Tuberculosis among Prison Inmates in Ethiopia, a Cross-Sectional Study
Source: PLoS One. 2015 Dec 7;10(12):e0144040. doi: 10.1371/journal.pone.0144040 (PMC4671540; doi:10.1371/journal.pone.0144040)

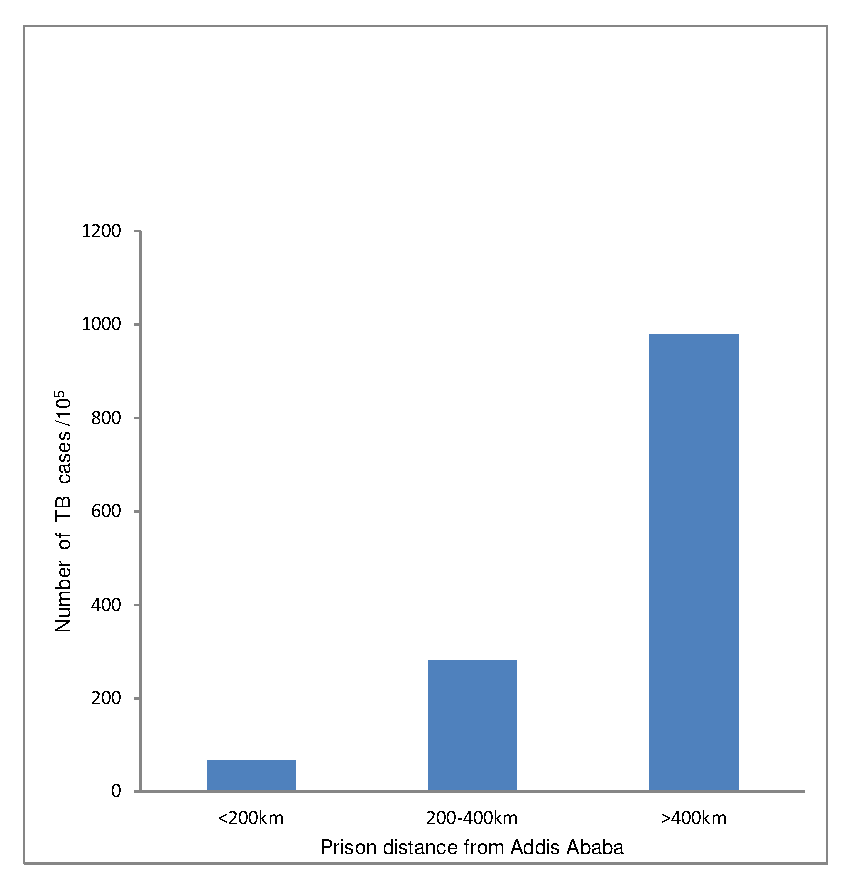

Supplement: S1 Fig — <200km from Addis Ababa: Ambo, Wolkite and Asella prisons, 200-400km from Addis Ababa: Shashemene, Nekemte, Sodo, Asebe Teferi/Chiro, Jimma´prisons, >400km from Addis Ababa: Bonga, Mizan, Dilla, Yabelo and Harar prison. (TIF) [file pone.0144040.s002.tif]
